# International Collaboration to Develop a Remote Monitoring Web App for COVID-19 Patients in Armenia: Design and Development With Agile Methodology

**DOI:** 10.2196/40110

**Published:** 2022-11-25

**Authors:** Abu Sikder, James Dickhoner, Lynn Kysh, Lusine Musheghyan, Shant Shekherdimian, Barry Levine, Juan Espinoza

**Affiliations:** 1 Innovation Studio Children's Hospital Los Angeles Los Angeles, CA United States; 2 Turpanjian College of Health Sciences American University of Armenia Yerevan Armenia; 3 Division of Pediatric Surgery University of California, Los Angeles Los Angeles, CA United States; 4 Department of Computer Science San Francisco State University San Francisco, CA United States; 5 Department of Pediatrics Keck School of Medicine University of Southern California Los Angeles, CA United States

**Keywords:** COVID-19, global health, software, mHealth, Armenia, web app, home monitoring, software development, human-centered design, remote monitoring, patient care

## Abstract

**Background:**

COVID-19 has led to over 500 million cases and 6.2 million deaths around the world. Low- and middle-income countries (LMICs) like Armenia face unique infrastructure, financial, and capacity challenges that in many cases result in worse outcomes. Health care facilities across Armenia experienced a shortage of resources, including hospital beds and oxygen, which was further exacerbated by the war with neighboring Azerbaijan. Without a framework for home-based care, health care facilities were severely strained by COVID-19 patients who had prolonged oxygen requirements but were otherwise clinically stable.

**Objective:**

This paper describes our approach to establishing an international collaboration to develop a web app to support home monitoring of patients with COVID-19 with persistent oxygen requirements.

**Methods:**

The app was developed using a rapid, coordinated, and collaborative approach involving an international group of clinicians, developers, and collaborators. Health screening, monitoring, and discharge forms were developed into a lightweight OpenMRS web app and customized for the local Armenian context.

**Results:**

The software was designed and developed over 2 months using human-centered design and agile sprints. Once live, 5087 patient records were created for 439 unique patients.

**Conclusions:**

This project suggests a promising framework for designing and implementing remote monitoring programs in LMICs, despite pandemic and geopolitical challenges.

## Introduction

The rapid transmissibility and pathogenicity of COVID-19 has led to over 500 million cases and 6.2 million deaths worldwide [1-4]. Health care facilities around the world have become overwhelmed through depletion of critical resources, such as hospital beds, mechanical ventilators, and key medications [5]. Low- and middle-income countries (LMICs) face unique infrastructure, financial, and capacity challenges that in many cases result in worse outcomes [6].

**Figure 1:**
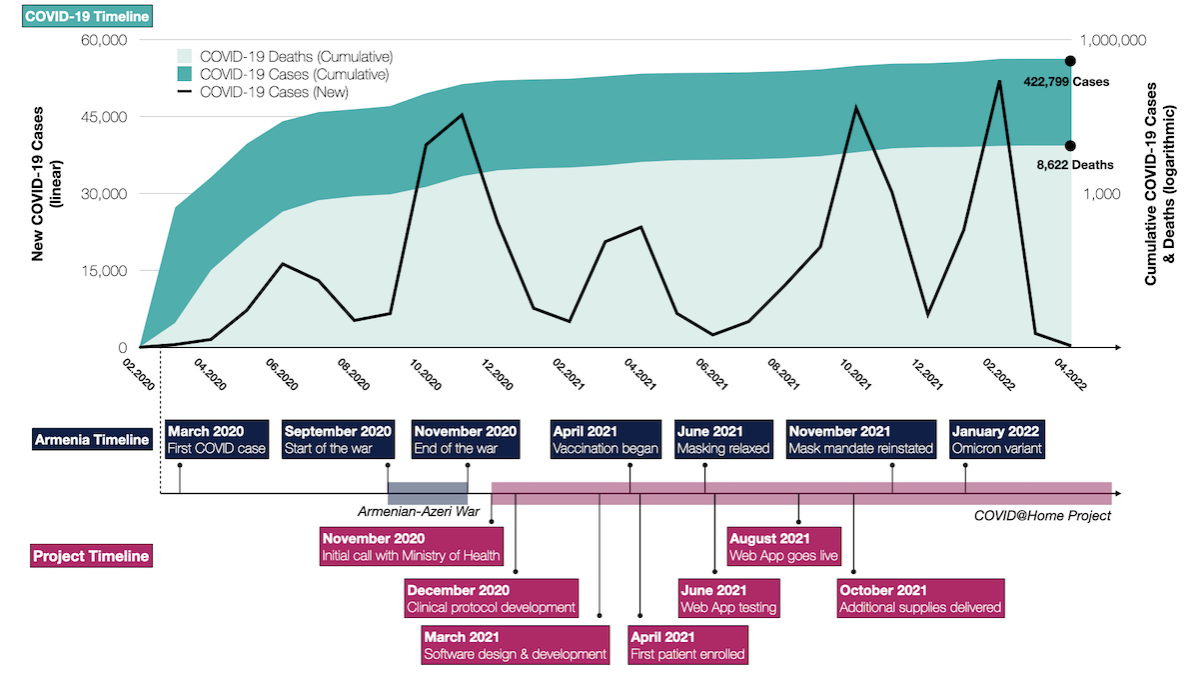
Composite timeline of events in Armenia related to the COVID@Home Project (data obtained from Our World in Data [10], which is licensed under Creative Commons Attribution 4.0 International License [11]).

Armenia, an LMIC situated in western Asia with a population of 2.98 million, had among the highest prevalence of COVID-19 for several months [1,7,8]. As a post-Soviet nation operating under the Semashko model’s influence, Armenia’s health care system is affected by significant fragmentation and specialization of hospitals [8]. The first COVID-19 case in Armenia was identified in March 2020, with numbers increasing substantially in the following months (Figure 1) [8]. There was a shortage of resources, including hospital beds and oxygen, that was further exacerbated by the war with Azerbaijan for the disputed Nagorno-Karabakh region from September to November 2020 [8,9]. The Armenian health care system faced immense pressure during this time, as health care facilities struggled to manage COVID-19 in addition to war casualties and other public health challenges [8]. Without a framework for home-based care, hospitals became crowded with patients with ongoing oxygen requirements who were otherwise clinically stable. This became a significant burden during the second wave of the pandemic in early 2021.

In response, Armenia’s Ministry of Health, the Turpanjian College of Health Sciences of the American University of Armenia, the University of California, Los Angeles (UCLA), and Children’s Hospital Los Angeles (CHLA) collaborated to develop a remote monitoring program to enable patients with prolonged oxygen requirements to be safely discharged home. Teams of clinicians, software developers, researchers, and administrative staff from both US- and Armenia-based institutions designed and implemented a home-based remote monitoring program (COVID@home) for low-risk patients tailored to the local Armenian context. This multinational, multidisciplinary collaboration was made possible by long-standing partnerships between these institutions focused on improving child health outcomes, preventive health screenings, supporting postgraduate medical education, implementing health technology innovation, and building local capacity [12-17]. Both UCLA and CHLA have existing institutional and grant-funded priorities to partner with Armenian institutions to improve health and health care delivery.

Remote patient monitoring programs have shown promise in managing low-risk patients while offloading the burden and use of critical resources in health care facilities. Although studies have adapted these programs to manage COVID-19 patients, the literature is limited on their utility and design, and none have been developed for Armenia. Due to cost, health care infrastructure requirements, and underdeveloped regulatory frameworks, there are many challenges with implementing and maintaining these systems in LMICs [15]. A promising and popular solution is OpenMRS, an open-source electronic health record (EHR) platform maintained by a global community of developers [18,19]. For this project, we customized an instance of OpenMRS and developed the COVID@Home web app to support our remote monitoring program in Armenia. Many software development methodologies exist, such as waterfall, spiral, V-shaped, and agile [20]. Due to the rapidly evolving nature of the COVID-19 pandemic, we leveraged an iterative, agile software development methodology in order to be responsive to the realities on the ground [20,21]. This paper describes our specific approach to designing and developing the web app using a collaborative, international, multidisciplinary team amidst unique pandemic and geopolitical challenges. A separate report covering the clinical protocol and outcomes is in preparation.

## Methods

### Program Overview

**Figure 2:**
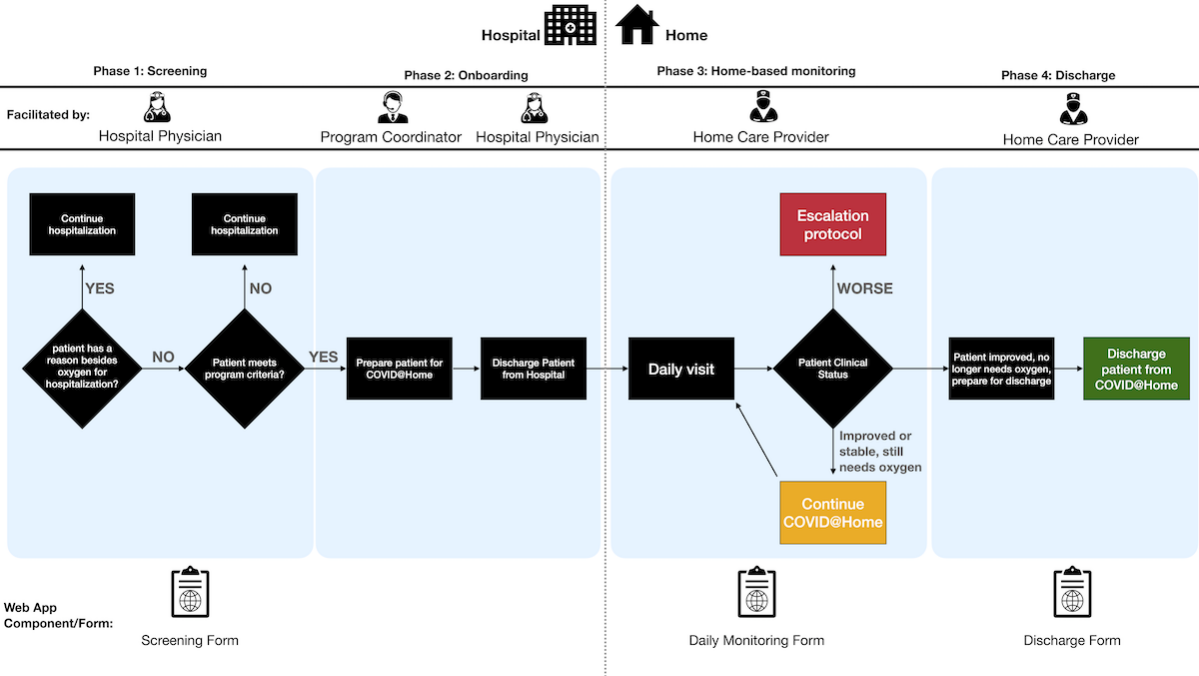
COVID@Home program overview and workflow with corresponding web app forms.

The COVID@Home program was designed to provide home monitoring of hospitalized patients with COVID-19 who had an ongoing oxygen requirement but were otherwise clinically stable. The two main components of the program were (1) inpatient screening and (2) at-home management, both of which were facilitated by the web app (Figure 2). Patient screening for program eligibility, which included a home safety evaluation, used the web app and was performed by a project nurse. If eligible, the patient was enrolled and consent was requested. Hospital discharge notes were made using the web app, and the patient was discharged with oxygen supplies and a pulse oximeter, along with appropriate education and instructions. Each day a home care provider (HCP) called the patient to check in on them and collect monitoring information using the web app. Once patients met specific clinical criteria, they were discharged from the program, the oxygen supplies were picked up from their home, and a discharge note was completed using the web app. Patients were transferred to the hospital if their condition worsened.

### Patient Population

The target patient population was hospitalized adult COVID-19 patients in Armenia. Clinically, patients were required to have stable vital signs (including blood pressure, heart rate, temperature, and breathing rate), stable glucose levels, and oxygen saturation >93% on no more than 10 L/min. Other eligibility criteria included access to a phone, a safe living environment, and access to electricity. The program was hosted in Yerevan with patients from 7 participating hospitals: Mikayelyan Institute of Surgery, Surb Grigor Lusavorich Medical Center, the National Center for Infectious Diseases, Avan Clinic of Surb Grigor Lusavorich Medical Center, Surb Astvatsamayr Medical Center, Scientific Center of Traumatology and Orthopaedy, and Erebuni Medical Center. Patients lived either in Yerevan or in close proximity to it (<40 km).

### Software Development Team

Development of the software involved 3 clinicians, 2 program administrators, 3 software developers, and 1 project manager. All team members resided in either Armenia or the United States.

### Ethics Approval

This study received internal review board approval (#CHLA-22-00028) from the CHLA.

### Software Development

#### Human-Centered Design

Human-centered design (HCD) is an empathetic problem-solving technique centered around the experiences and needs of people [22]. It is often used as an iterative and collaborative process that intimately involves stakeholders and end users [22]. Our process began with identifying and interviewing critical stakeholders and users of the tool, ranging from administrators and leaders to frontline clinical staff. By engaging these individuals early on, we were able to develop trust and gather their feedback at each stage of the development process. Our users identified specific problems and their needs for the proposed tool. We completed several rounds of ideation using low-fidelity mockups for rapid prototyping and gathered feedback from our users to inform the development of our tool. Once the research team and stakeholders arrived at a meaningful design, a functional version was released. We conducted additional interviews with the program managers to ensure the tool was well integrated into our end users’ workflows. The app was then localized by professional Armenian translators for both proper language use and cultural competency. Finally, after deploying the tool, we completed usability testing with our users to further refine the tool and ensure it was meeting their needs.

#### Project Management

The web app was developed using an iterative, agile methodology based on weekly sprint sessions with stakeholders. Each week the core team (program administrators, researchers, and software developers) met over Zoom (Zoom Video Communications) to review and set tasks involving the creation of new features, making updates, fixing bugs, and testing the software. Action items were stored as tickets in JIRA, a software management program (Atlassian). Updates were recommended by core members and end users after testing and reviewing the features or code. Stakeholders were actively consulted after milestones to obtain feedback and improve on the design and functionality of the software as needed.

#### Tools and Technology

OpenMRS is a web-based application built using Spring MVC (a web app framework) that uses a custom application programming interface (API) to interact with the data model [23]. It relies on a concept dictionary based on the Columbia International eHealth Laboratory for storing and retrieving patient data [24]. Another important design feature of OpenMRS is the use of modules that have full access to the OpenMRS system, including the database, API, and front end. The modules are used to expand or customize features of the core application.

#### Architecture

**Figure 3:**
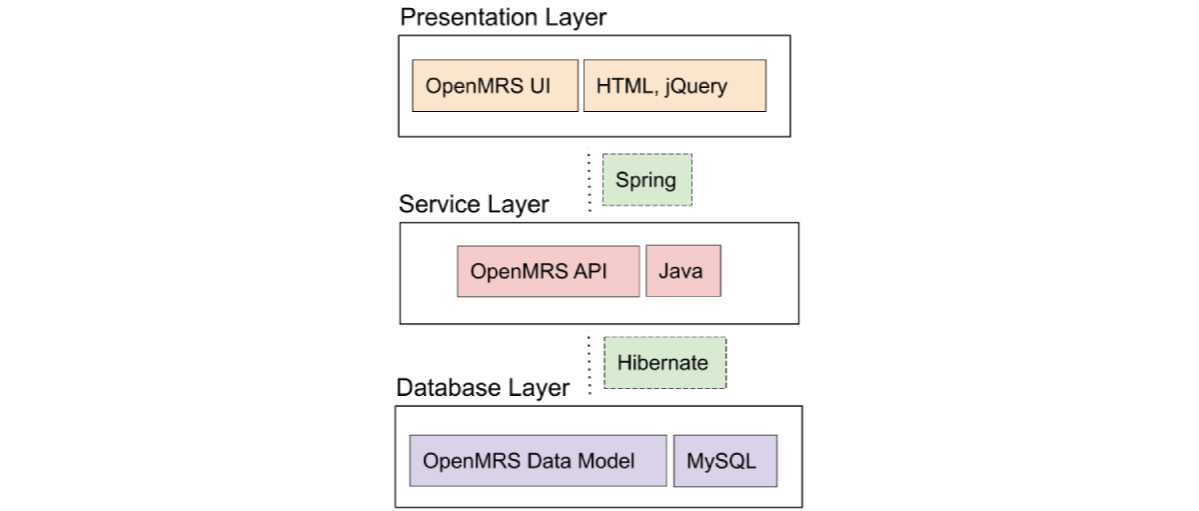
Architecture of the COVID@home OpenMRS platform. API: application programming interface; UI: user interface.

The web app architecture includes a presentation layer (PL) with HTML, jQuery, and other front-end technologies (eg, React), a service layer (SL) with Java, and a database layer (DL) with MySQL (Figure 3). Spring MVC helps interface the PL and SL by following the model-view-controller design pattern [25,26]. Hibernate is used as an object relational mapper [27]. All the various layers can be accessed and modified using modules via direct access to the API. The web app also uses custom-built OpenMRS add-on modules that interact with all 3 layers of the core system. The modules provide additional web pages with associated SL code and database tables.

### Data Collected

#### Forms

Three forms were developed by the clinical team and integrated into the web app: (1) a screening form completed by hospital and program staff, (2) a daily monitoring form completed by the HCPs, and (3) a discharge form completed by the HCPs once a patient was discharged from the program. Each form contained a distinct set of questions about the participant’s health, demographics, vital signs, and clinical history. Both English and Armenian versions of the forms were made available.

#### Workflow

The HCPs updated the forms directly in the web app for screening, health monitoring, and discharge. In addition, hard copies were made available, and the information was re-entered manually in the web app system at a later time. The data were then made available via reports created within the OpenMRS system.

## Results

### HCD Process

The HCD process lasted 2 months, with design meetings involving US- and Armenian-based physicians, engineers, and project managers that were hosted over Zoom. Deliverables included a complete wireframe, a spreadsheet with Armenian language translations, and a document with user acceptance testing (UAT) feedback. Deliverables were reviewed by Armenian clinicians for suitability for use in Armenia. Comments from the UAT helped improve the user interface and user experience, such as by adding clicks, improving navigation, and adding a function to validate form input. Hard-copy backups were included at the suggestion of users.

### Development Logistics

Six biweekly meetings were held with the software developers to finalize the platform. Meeting times were adjusted to better accommodate the fluctuating and busy schedules of the clinical staff. Using the initial deliverables from HCD, the software developers created a demonstration version of the app that was validated by stakeholders through trial and error. Over 200 JIRA tickets were completed during development and testing. The final version was rolled out to end users and implemented by local stakeholders with ancillary services from US colleagues.

### Software Components: Features and Functionality

**Figure 4:**
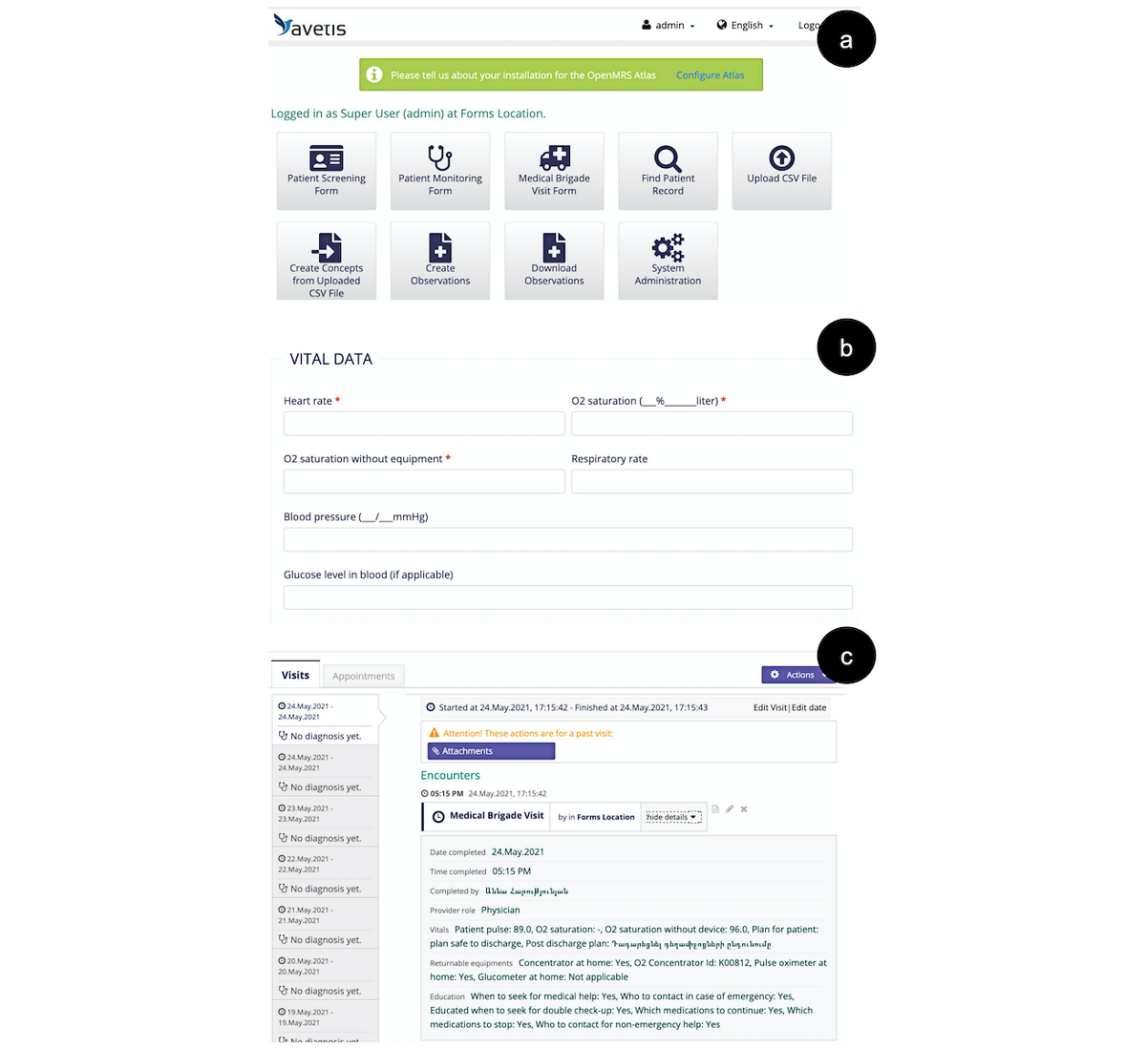
The OpenMRS front end for (a) COVID@home, with forms, modules, and admin options; (b) the form data entry page; and (c) an example of a completed patient record.

Five customized web pages were created: a login page, a home page, and 3 screening forms (Figure 4). Four OpenMRS modules were developed for inserting comma-separated value (CSV) data from hard-copy forms, exporting form data, editing participant status, and finding records. There were more than 100 new OpenMRS concepts created to accommodate each data point in both Armenian and English. Concepts were integrated using 2 Groovy scripts: CSV to concepts and CSV to observations. Custom drug classes were also included using 2 Groovy scripts: CSV to drug classes and concept from drug classes. A total of 52% of the code was written in Java and 47% in Groovy.

### Platform Implementation and Utilization

The COVID@Home program started enrolling patients in April 2021 (Figure 1). During the first few months, while the web app was still being developed, data were tracked in an online form. The web app went live in August 2021, and all records prior to that point were batch imported into the web app platform. Between April 2021 and March 2022, 439 unique patients completed 5087 forms using the web app. A majority of the forms were from daily monitoring (4430/5087, 87%), followed by screening (439/5087, 9%) and discharge (218/5087, 4%) forms. A total of 14 staff members were initially trained on how to use the web app and complete the forms. The staff received informal support from the technical team through tutorials on logging in, resetting passwords, and using the web app. Paper forms were used to collect data as needed, such as when computers or support staff became unavailable. The paper forms were later entered into the web app database. The software worked as expected, and no critical bugs or issues were reported.

## Discussion

### Principal Findings

Our international, multidisciplinary partnership successfully developed and implemented a free, open-source web app to remotely monitor COVID-19 patients in Armenia at home by leveraging HCD and an agile software development methodology. Developing software with international colleagues required attention to logistics and strategy. Active involvement with local stakeholders in Armenia was a priority. Due to substantial differences in time zones, virtual meetings were held early in the morning in the United States (Pacific time), corresponding to evening in Armenia. Additionally, program administrators from the United States visited colleagues in Armenia to establish rapport and facilitate stakeholder buy-in for the project when travel restrictions were relaxed. We explicitly used an HCD approach to ensure that the web app was linguistically and culturally relevant and responsive to the local technical environment and workflows. An important requirement for adapting the software for the end user was the integration of language translations for all components of the web app. We contracted the software development to a local team in Armenia and communicated with local stakeholders, which allowed us to test and iterate the web app more effectively.

We searched PubMed for publications describing COVID-19 patients who were discharged home with supplemental oxygen therapy and their remote monitoring and management using mobile or web apps with the following key words and Medical Subject Headings (MeSH) terms: (COVID-19/therapy[Mesh]) AND (Telemedicine[Mesh] OR Home Care Services[Mesh] OR Telemetry[Mesh] OR Patient Discharge[Mesh] OR telemedicine OR home OR telemetry OR remote monitor* OR virtual) AND (Oxygen Inhalation Therapy[Mesh] OR oxygen). We identified a total of 5 studies, which took place in the Netherlands (n=1), United States (n=2), United Kingdom (n=1), and Egypt (n=1) [28-32]. Aside from the Egyptian study, all the studies took place in high-income countries (HICs). This imbalance reflects the technical, infrastructural, and financial health care divide between HICs and LMICs. All 5 studies had 2 common features: collection of vital signs and communication via telephone [28-32]. Progress assessment and vital signs, such as oxygen saturation, heart rate, temperature, and blood pressure, were captured by all the studies and then stored in electronic databases, not unlike our current project. Capturing health data in electronic databases helps support research, collaboration, and quality improvement [33]. Although all the studies involved a mobile app (for education, assessment, or monitoring vital signs), they all relied on communication with the clinical team via phone calls [28-32]. Our program operated similarly, which supports the utility of leveraging standard forms of communication for remote monitoring. Three of the 5 studies conducted patient satisfaction surveys, with all of them reporting 94% or greater satisfaction with the remote monitoring program and emphasizing the benefit and utility to patients [28,29,32]. With respect to the development of these programs, only 1 described the process in detail [30]. The Atrium Health Hospital at Home (AH-HaH) program was designed and implemented rapidly in the United States with the help of numerous stakeholders with backgrounds including clinical medicine, administration, research, technology, and innovation [30]. This mirrors our process and reiterates the multidisciplinary nature of developing home-based clinical programs supported by technology. However, the AH-HaH program was implemented using existing infrastructure and resources that are not often available in LMICs [30]. Evidently, our program in Armenia was developed with a limited amount of existing infrastructure and operational resources. Other remote monitoring programs have also commented on the importance of a flexible and nimble development framework due to changes in COVID-19 guidelines, an observation that reflected our experience in Armenia as well [34].

The main strength of our project was the rapid, coordinated, and collaborative approach. With logistical uncertainty, limited knowledge, and the evolution of COVID-19 and its variants, it was important to quickly and responsively develop health screening and monitoring forms with the most recent available knowledge. This required updating form questions, the criteria, and form fields as more evidence became available. In turn, the software development cycle reflected this process and required updating code and functionality simultaneously. The conflict with Azerbaijan in 2020 had further strained the Armenian health care system, so the iterative, agile methodology allowed the team to be flexible and responsive to evolving capacity. Other strengths were the reliance on scientific evidence and the involvement of clinicians in the development process. Although case studies and literature on COVID-19 were available, the novelty of the disease and evolution of new strains led to uncertainty. Knowledge of the disease within the context of Armenia was even more limited. However, frontline workers in Armenia helped design the clinical protocols and data collection forms and were informed by their experiences and the best available evidence. This project was truly a collaborative effort, involving individuals of diverse professional experiences, cultures, disciplines, and knowledge. Each feature and change was tested by stakeholders and end users, who provided guidance on adapting the system for local use, such as suggesting language changes and workflow updates. The COVID@Home program would not have been possible without a multidisciplinary and collaborative team of clinicians, software developers, researchers, and local stakeholders. Finally, COVID@Home set a precedent for active data collection, reporting, monitoring, and outcome evaluations outside of a research setting, which is rarely done in Armenia.

A significant limitation of our approach was the reliance on hard-copy forms, which affected the quality of the data. At various points during the project, computers were only intermittently available for data entry. This resulted in patient data being recorded on paper forms instead of the web app. By the time this was brought to the attention of the project team, hundreds of patient records had been created using paper forms. Dedicated computers were provided for data entry, web app access, and digitizing the paper forms, but this resulted in several records with nonstandardized or missing data. During this time, the software continued to operate as expected. A general challenge across many global health partnerships is the long-term sustainability of a project [35]. This is a critical component of global health partnerships and is built into all of our collaborations in Armenia. For this particular project, the total number of patients enrolled in the program has drastically decreased since July 2022 as COVID-19 hospitalizations have decreased, and the plan from the Ministry of Health is to sunset this program. The technical infrastructure is maintained by a local team that supports multiple grant-funded projects, and they are committed to continue operating the existing system. As this was the first time a home health-monitoring program was used by the Ministry of Health, the ministry is currently evaluating whether to expand this approach to other use cases.

A key lesson learned from this experience was the importance of clear communication and training for all users, particularly the frontline HCPs. A more thorough needs and capacity assessment likely would have detected the issue of paper-based data collection; although the project team asked if a computer was available, additional follow-up questions may have revealed that there were limitations affecting who could access the computer, how often, and how often there were interruptions to that access. Prospective utilization monitoring might also have detected that new records were not being created, prompting further investigation. As an operational initiative leveraging existing staff and resources, the project did not operate in a vacuum, and was affected by broader factors in the health system: lack of human resources, lack of planning, and lack of proper equipment. For example, in one instance, the project coordinator was reassigned, leaving frontline staff with no support for several weeks. The development of paper forms played an important and pragmatic role in the success of the program, though once staff transitioned to paper forms due to computer access issues, it was difficult to shift the workflow back to the web app. In a less chaotic environment, not having paper forms at all may help increase the speed of adoption and prevent parallel workflows. Future projects will need to better evaluate cultural, sociotechnological, and logistical barriers to technology adoption in busy health care settings.

### Conclusion

COVID-19 has caused tremendous clinical and logistical challenges around the globe, particularly in LMICs. Our experience in Armenia demonstrates that an international collaboration can leverage an agile methodology to rapidly develop software needed to support a home monitoring program for patients with prolonged oxygen requirements secondary to COVID-19 using free, open-source software. This project can serve as an example for at-home care in resource-limited settings like Armenia and other LMICs. Successful implementation requires a thorough needs assessment, clear and open communication, and prospective utilization monitoring to identify and address barriers.

## Abbreviations

AH-HaH: Atrium Health Hospital at Home
API: application programming interface
CHLA: Children’s Hospital Los Angeles
CSV: comma-separated value
DL: database layer
EHR: electronic health record
HCD: human-centered design
HCP: home care provider
HIC: high-income countries
LMIC: low- and middle-income country
MeSH: Medical Subject Headings
PL: presentation layer
SL: service layer
UAT: user acceptance testing
UCLA: University of California, Los Angeles
